# Distance-aware framework on molecular engineering of electrocatalytic interfaces

**DOI:** 10.1039/d6sc05420j

**Published:** 2026-08-24

**Authors:** Zhihao Wang, Xu Zhang, Lin He, Zhimin Chen, Zhiyu Ren

**Affiliations:** a School of Chemistry and Materials Science, Key Laboratory of Functional Inorganic Material Chemistry (Ministry of Education), National Center for International Research on Catalytic Technology (Ministry of Science and Technology), Heilongjiang University Harbin P. R. China zmchen@hlju.edu.cn zyren@hlju.edu.cn

## Abstract

Molecular engineering provides effective strategies for optimizing electrocatalytic interfaces; however, a physically grounded conceptual framework that provides an intuitive picture of molecular engineering is still lacking. This perspective proposes a distance-aware framework that decouples molecular modification effects into three distinct spatial zones relative to the active site, each characterized by different dominant interactions. Inner-layer engineering targets the angstrom-scale region immediately adjacent to the surface, where modifiers directly participate in charge redistribution and intermediate stabilization to tailor surface energetics. Intermediate-layer modulation governs the local microenvironment, mediating reactivity through local concentration fields, hydrogen-bond networks, and electrostatic double-layer effects. Outer-layer engineering operates at the bulk interface, controlling macroscopic wettability, interfacial shielding, and long-term structural stability. By establishing this spatially resolved and decoupled perspective, we propose a distance-based framework to systematically organize and interpret the fragmented modification phenomena, thereby providing a physically grounded structured framework for organizing molecular engineering strategies.

## Abstract 2

A distance-aware framework organizes molecular modifications at catalytic interfaces according to their proximity to active sites, providing a physically intuitive perspective to understand spatially distinct interfacial regulation strategies.

## Graphical abstract

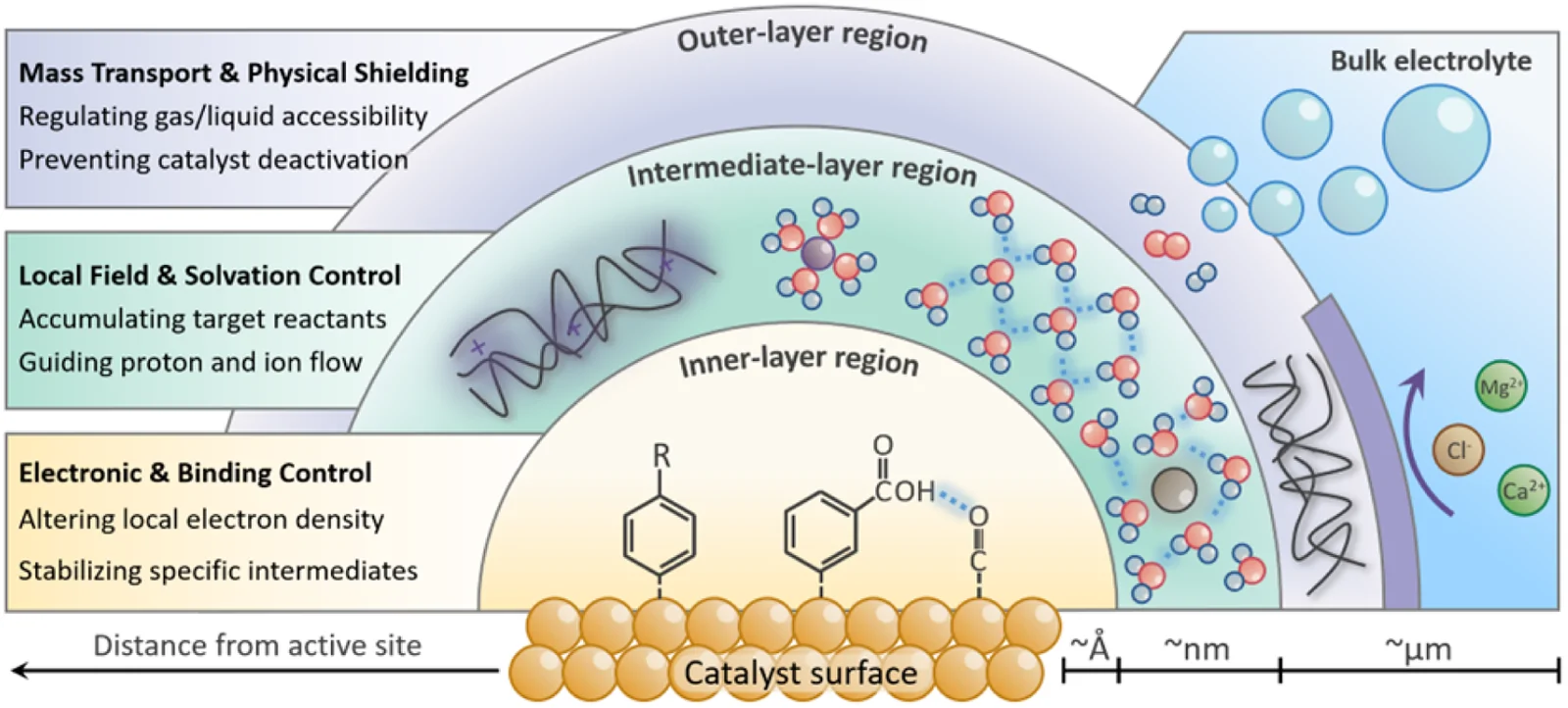

## 1. Introduction

The rapid depletion of conventional fossil fuels is inevitably accompanied by severe energy shortages and environmental pollution.^1,2^ With the continued global push toward energy transition and carbon neutrality, the development of clean-energy production, conversion, and storage technologies is expected to provide critical support for future energy supply. As a result, electrochemical energy-conversion processes, including water (H_2_O) electrolysis,^2^ carbon dioxide (CO_2_) reduction,^3,4^ nitrogen/nitrate (N_2_/NO_3_^−^) reduction,^5,6^ and organic oxidation,^7,8^ have become central topics in catalysis research. These reactions generally involve coupled multi-electron/multi-proton transfer, together with complex intermediate evolution and interfacial mass transport. This complexity imposes increasingly stringent requirements on catalyst activity, selectivity, and stability under realistic operating conditions.^9,10^ Conventional optimization strategies, such as compositional regulation, facet engineering, defect construction, and heterostructure design,^11–13^ have delivered substantial progress in improving intrinsic catalytic activity. However, their regulation is largely confined to the catalyst bulk or near-surface region and is increasingly approaching methodological limits, making it difficult to further overcome existing performance bottlenecks.

Against this background, the paradigm of electrocatalysis research is shifting from the isolated modulation of intrinsic activity toward cross-scale and subtle external modulation of the catalytic interface, with the aim of co-optimizing reaction pathways and interfacial environments.^14–16^ Owing to their molecular programmability, functional diversity, and dynamic responsiveness, molecular functionalization has emerged as a frontier direction in interfacial engineering. By introducing organic molecules, ligands, supramolecular architectures, or polymer layers onto inorganic catalyst surfaces, it becomes possible to achieve forms of precise regulation that are difficult to realize in conventional inorganic systems, while preserving the structural stability of the inorganic scaffold.^17,18^ The effectiveness of this strategy has been demonstrated across a wide range of electrocatalytic systems. In the past five years alone, several hundred studies on molecular modification have been reported, and this number continues to rise.

This sustained increase underscores the rising importance and substantial potential of molecular engineering in electrocatalysis. Several reviews have already summarized progress in this field by classifying systems according to specific reactions or material categories and discussing the interactions between organic interfaces and electrocatalytic processes.^19–21^ However, classification schemes that lack a clear physical intuition often fail to capture the general principles underlying molecular modification, thus limiting the systematic design and rational development of molecular-engineering strategies. A more fundamental classification framework is therefore needed to systematically reorganize molecular modification from a physically grounded perspective, thereby offering a more intuitive understanding of molecular engineering.

**Fig. 1:**
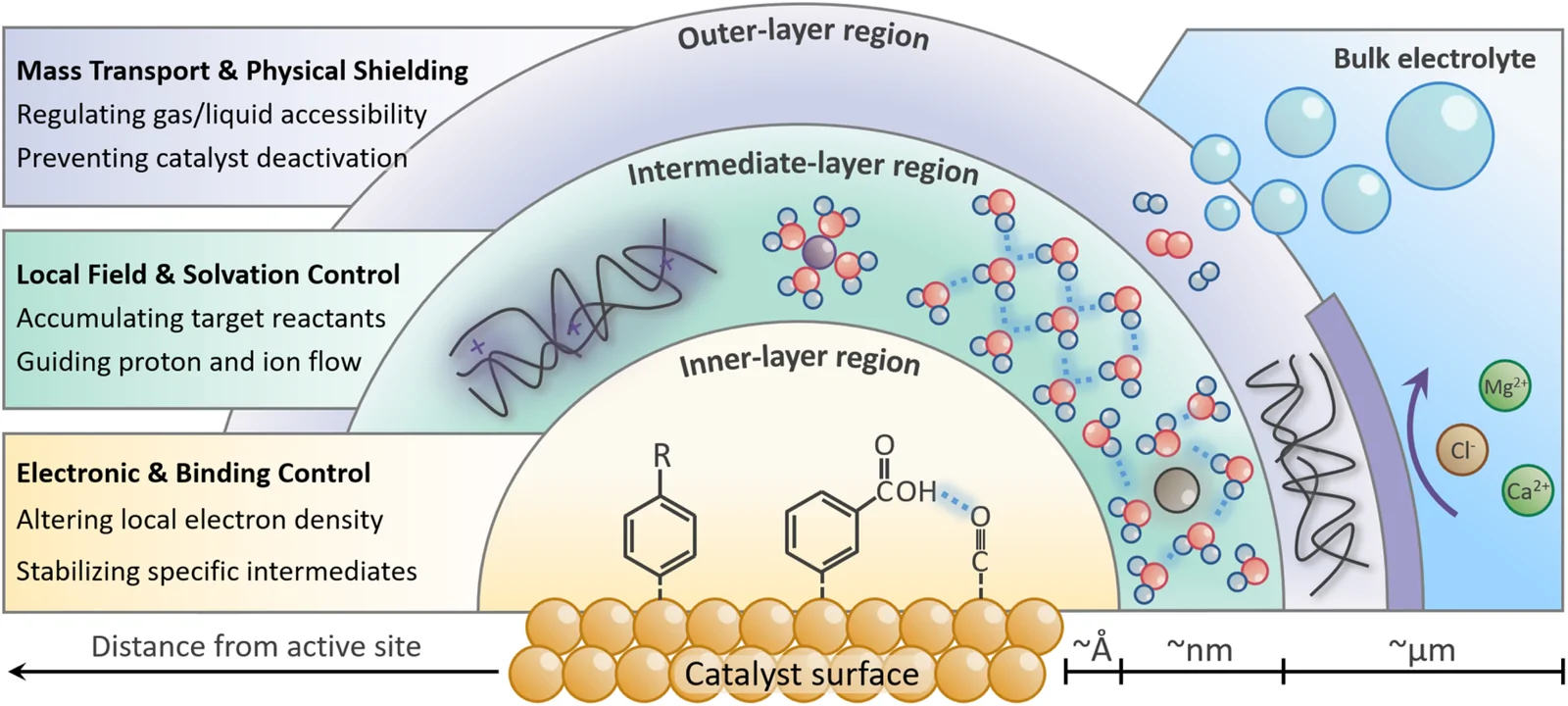
Schematic illustration of the distance-aware framework for molecular engineering at electrocatalytic interfaces. Based on the spatial proximity to the catalytic active sites, the regulatory mechanisms of modifying molecules are decoupled into three spatial zones: the inner-layer region for direct regulation of intrinsic surface energetics (∼Å), the intermediate-layer region for microenvironmental mediation (∼nm), and the outer-layer region for macroscopic mass transport and structural shielding (∼µm).

Interestingly, the influence of surface modifiers on interfacial electrocatalytic processes exhibits a pronounced spatial dependence. Their dominant effects and underlying mechanisms vary systematically with the physical distance between the modifier molecules and the catalytic active sites. In regions close to the active site, modifiers are more likely to directly participate in electronic-structure regulation and reaction-barrier tuning. In contrast, in regions farther from the active site, their effects are more often expressed through reshaping of the interfacial environment and macroscopic boundary conditions. This distance dependence provides an entry point for understanding both the mechanism and the applicability of molecular engineering (Fig. 1).

Based on this consideration, we propose a distance-aware framework centered on the spatial proximity between modifying molecules and catalytic active sites (Table 1). According to the interaction distance, the effects of modifiers can be heuristically decomposed into three interconnected spatial regions extending progressively away from the active site, each characterized by distinct dominant interactions and regulatory mechanisms. The inner-layer region corresponds to the angstrom-scale (Å) region immediately adjacent to the catalyst surface, where electronic-structure effects are most prominent. At this scale, modifiers can induce charge redistribution, alter orbital interactions, and influence the adsorption and stabilization of key intermediates, thereby tuning the intrinsic reaction energetics. The intermediate-layer region spans the dynamic Debye interface on the nanometer-scale (nm) and is primarily described by statistical thermodynamics.^22^ Here, modifiers regulate local concentration fields, reorganize hydrogen-bond network structures, and modulate the local dielectric environment. These effects collectively influence interfacial reaction kinetics and catalytic selectivity. The outer-layer region extends into the micrometer-scale (µm) domain and beyond, where continuum transport and fluid-dynamic effects become increasingly important. In this region, modifiers primarily shape the macroscopic reaction environment by tuning wettability, regulating mass transport, providing interfacial protection, and buffering structural evolution, thereby contributing to mass transfer and catalyst durability.^23^ Furthermore, by linking the functions of each spatial region to appropriate experimental characterization techniques, layer-specific effects can be systematically attributed, thereby providing a more intuitive understanding of molecular interface design. Cross-layer coupling among different spatial regions and the practical limits of molecular modification are also discussed. Together, this spatially layered description integrates previously discrete molecular-regulation phenomena into a distance-aware framework of the interfacial control. Such a framework provides a more coherent interpretation of distinct regulatory mechanisms and offers a new perspective on molecular engineering for the development of next-generation high-performance electrocatalytic systems.

**Table 1:** Definition and characteristics of the distance-aware framework at electrocatalytic interfaces

Layer	Spatial scale	Key features	Dominant interactions	Resulting effect
Inner	∼Å	Charge redistribution	Inductive effects	Intrinsic energetics
			Orbital coupling	Thermodynamics
			Intermediate stabilization	
Intermediate	∼nm	Noncovalent interactions	Local concentration fields	Reaction kinetics
			H-bond network reconfiguration	Pathway selectivity
			Double-layer reshaping	
Outer	∼µm	Species accessibility	Wettability control	Mass transport
			Interfacial shielding	Ion tolerance

## 2. Inner-layer engineering: direct regulation of surface energetics

At the innermost region of the electrocatalytic interface, modifying molecules are in sub-nanometer-scale direct contact with active sites. This short spatial distance allows the modifiers to directly participate in charge redistribution on the catalyst surface, orbital coupling, and stabilization of key intermediates. By finely reshaping the reaction energy landscape, such modifiers can directly influence adsorption strength, transition-state stability, and the competitive relationship among reaction pathways. This tuning strategy can optimize selectivity and kinetics without altering the primary catalyst composition, but it is typically accompanied by strong structural sensitivity.

### 2.1 Electronic structure

**Fig. 2:**
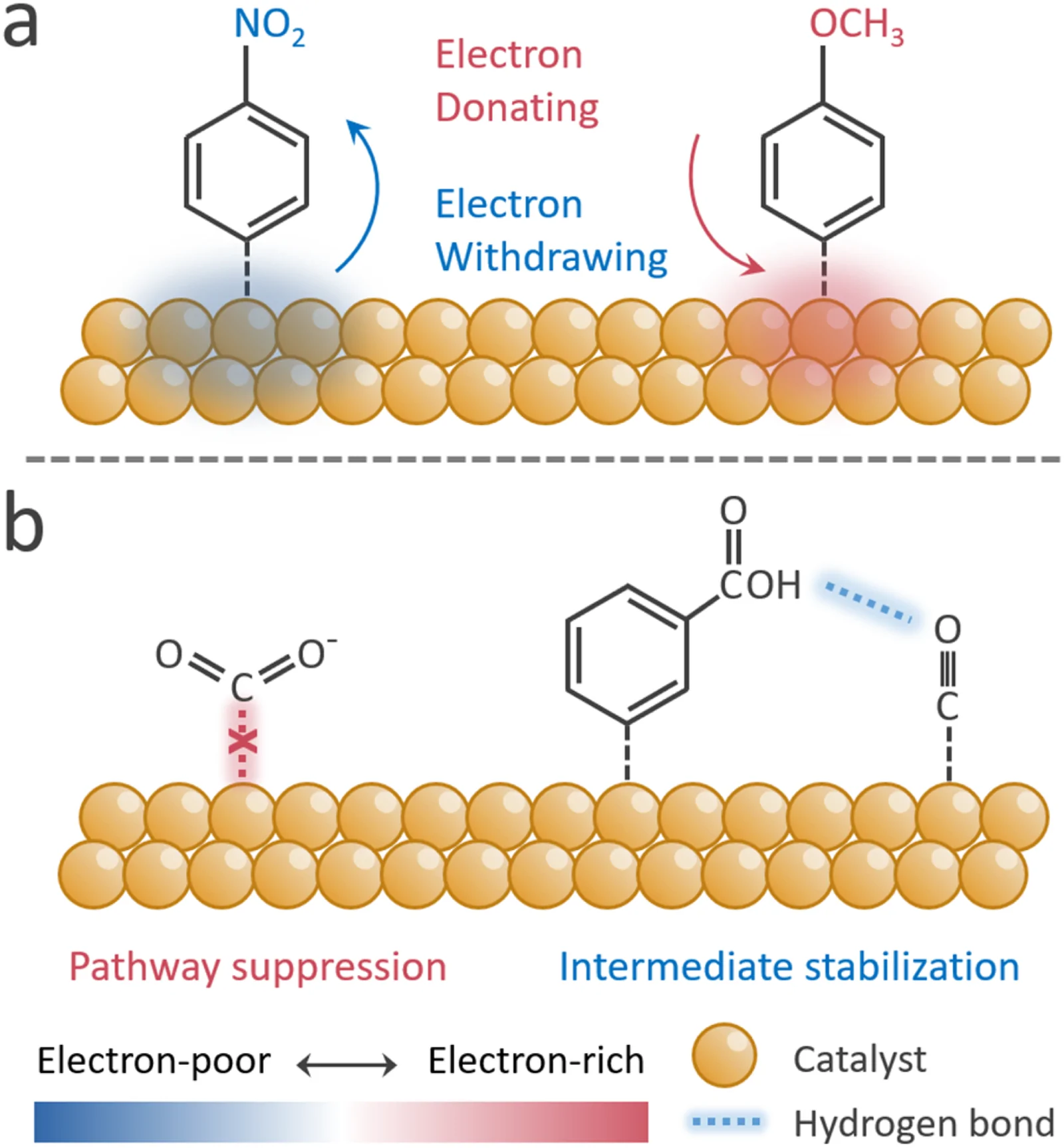
Inner-layer engineering for direct regulation of surface energetics. (a) Modulation of local electronic structures. Electron-withdrawing or electron-donating modifiers directly alter the surface electron density (creating electron-deficient or electron-rich states), thereby tuning the binding strength of adsorbates. (b) Steering of reaction pathways. Anchored modifying molecules can precisely dictate reaction selectivity by either suppressing competing pathways (left) or stabilizing key intermediates *via* direct noncovalent interactions, such as hydrogen bonding (right).

Surface ligands directly alter charge distribution in the inner layer (Fig. 2a). Through electron-donating or electron-withdrawing effects, modifiers can change local electronic states at the surface and influence key intermediates. For example, strongly electron-withdrawing molecules can extract electron density from metal surfaces, rendering certain sites more electron-deficient, weakening the binding of adsorbates, or promoting surface reconstruction. Pentafluorophenyl groups (–C_6_F_5_), as a case, markedly decrease the electron density of surface Ni sites, accelerate charge-transfer kinetics, and promote structural reconstruction into highly active γ-NiOOH for oxygen evolution.^24^ Consistently, electron-withdrawing fullerol-amine species induce electron-deficient states on Pd metallene surfaces; this specific electronic reconstruction favors the adsorption of electron-rich hydroxyl species and boosts ethanol oxidation activity.^25^ Conversely, certain ligands can stabilize transient oxidation states that are crucial for downstream multi-carbon pathways. For instance, citrate coordination on Cu_2_O promotes electron transfer from the metal to the carboxylate, thus helping preserve Cu^+^ species under reductive conditions and favoring multi-carbon product formation.^26^

Beyond charge redistribution, modifying molecules can also affect the band structure and orbital-hybridization state of catalysts, more directly altering the bonding characteristics of active sites. For instance, polyethylenimine (PEI) donates electrons to PdRh surfaces, shifts the d-band center downward, lowers the barrier for the *OOH → *O step, and changes the oxygen reduction reaction (ORR) pathway from an associative to a dissociative route.^27^ Similar orbital modulation has also been reported in complex oxides and high-entropy materials. Strong interactions between ionic liquids and Ba_0.5_Sr_0.5_Co_0.8_Fe_0.2_O_3−*δ*_ (BSCF) oxidize surface Co and Fe species, optimize eg orbital filling, and raise the O 2p band center to strengthen metal 3d/O 2p hybridization and enhancing ORR activity.^28^ In addition, electron-donating pyrrolidone groups introduced onto K(CoMnFeNiCr)F fluorides can tailor the intrinsic band structure and improve conductivity, thereby facilitating electron transport and OH^−^ adsorption.

Inner-layer molecular engineering relies strongly on precise matching with the surface. The intrinsic properties, anchoring mode, and conformation of a modifier all affect the degree of orbital coupling with the catalyst. A typical example is that when chiral helicene molecules (such as thiadiazole-[7]helicene) are used to modify a NiO_*x*_ electrode, different enantiomers (*P*- *vs. M*-) lead to differences in oxygen evolution reaction activity, demonstrating the strong structural sensitivity of molecular modification.^29^ In Ag nanocubes modified with a series of arylthiol ligands bearing different substituents, 4-(methylthio)benzaldehyde (MTBA)-modified Ag exhibits the highest Ag^+^/Ag^0^ ratio, optimizing *NO_3_ adsorption/dissociation and H_2_O activation and consequently delivering the most favorable nitrate-reduction thermodynamics. This example highlights how even subtle variations in modifier structure can lead to substantially different regulatory effects. Nevertheless, some studies have begun to establish clearer relationships between molecular structure and electronic effects. For instance, when Ru nanoparticles are modified with phenylacetylene derivatives bearing different para substituents, a linear correlation is observed between the Hammett substituent constant (*σ*) and hydrogen evolution reaction (HER) activity, suggesting that the electron-donating/-withdrawing capability of substituents will be useful for predicting surface electronic states and catalytic behavior.^30^ A quantitative understanding of the electronic effects introduced by molecular modifiers can help inform the rational design of inner-layer molecular regulation strategies.

### 2.2 Reaction intermediate

Whereas electronic-structure modulation primarily reshapes reaction thermodynamics, intermediate modulation acts more directly on key nodes along the reaction dynamics pathway (Fig. 2b). Through hydrogen bonding, electrostatic interactions, or steric constraints, modifying molecules can alter the formation, residence, and conversion of nearby intermediates, which govern selectivity pathway barriers are often reduced through stabilization of target intermediates. For example, tannic acid can simultaneously stabilize Cu^*δ*+^ species and form hydrogen bonds with *COH, enabling enhanced CO-dimerization related processes and improving C_2_H_4_ production.^31^ Likewise, in ORR systems, 2,6-diacetylpyridine promotes proton coupling with oxygen intermediates through electrostatic interactions, making the four-electron pathway more favorable.^32^

Molecular modifiers do not necessarily achieve selectivity control only by stabilizing desired intermediates; they can also do so by destabilizing competing species. For instance, pyridine molecules anchored on Cu interact with specific intermediates through the N atom, thermodynamically destabilizing them and thereby blocking the CO-formation pathway while redirecting the reaction toward formate.^33^ Suppressing poisoning can also be effective, as demonstrated by melamine acting as a surface blocker to selectively prevent sulfate-anion poisoning on Pt(111); this targeted suppression significantly accelerates ORR activity in sulfuric acid without markedly compromising oxygen-reduction kinetics.^34^

For reactions involving multiple reactants or intermediates, surface ligands often influence catalytic behaviour through the reconfiguration of the reaction network. By selectively promoting or suppressing the surface coverage of different intermediates, they alter the balance among competing elementary steps and consequently reshape reaction pathways and product distributions. As a case, thiophenol modification on Cu lowers the overall surface CO coverage, promotes *CHO formation, and suppresses the CO dimerization required for multi-carbon products, thus enhancing methane selectivity.^35^ In addition, the introduction of specific ligands (–SCN, –CN, and –NH_2_R) can simultaneously induce the formation of Cu^+^ species and suppress competing proton adsorption, tuning to the binding energies of *OCHO and *CO and steering product selectivity toward formate, ethylene, or C_1_ mixtures.^36^ Similarly, ionic liquids such as [Bmim][PF_6_] can simultaneously alter intermediate distributions and barrier heights, shifting products from C_2_H_4_ to CH_4_.^37^ Accordingly, molecular modifiers redistribute the energies and coverages of intermediates across multiple adjacent elementary steps.

Overall, inner-layer engineering represents the most proximal form of intrinsic regulation around active sites. It is often the most direct route for tuning adsorption strength and reaction pathways. At the same time, it is also the most constrained by the precise matching among molecular conformation, anchoring mode, and surface structure. In other words, inner-layer regulation generally requires precise loading and is accompanied by the strongest structural sensitivity.

## 3. Intermediate-layer engineering: microenvironmental mediation of interfacial reactivity

The intermediate layer serves as the transitional region between the catalyst surface and the bulk electrolyte. Here, modifying molecules shape the microenvironment of the reaction interface mainly through noncovalent interactions. Their functions can be broadly divided into three categories: orchestrating the distribution of reactants and intermediates, reorganizing interfacial water networks, and altering the potential distribution and ionic environment within the electrical double layer. Thus, intermediate-layer modulation reconstructs the reaction microenvironment over a more flexible spatial range to control species transport, enrichment, and coupling.

### 3.1 Local concentration fields

**Fig. 3:**
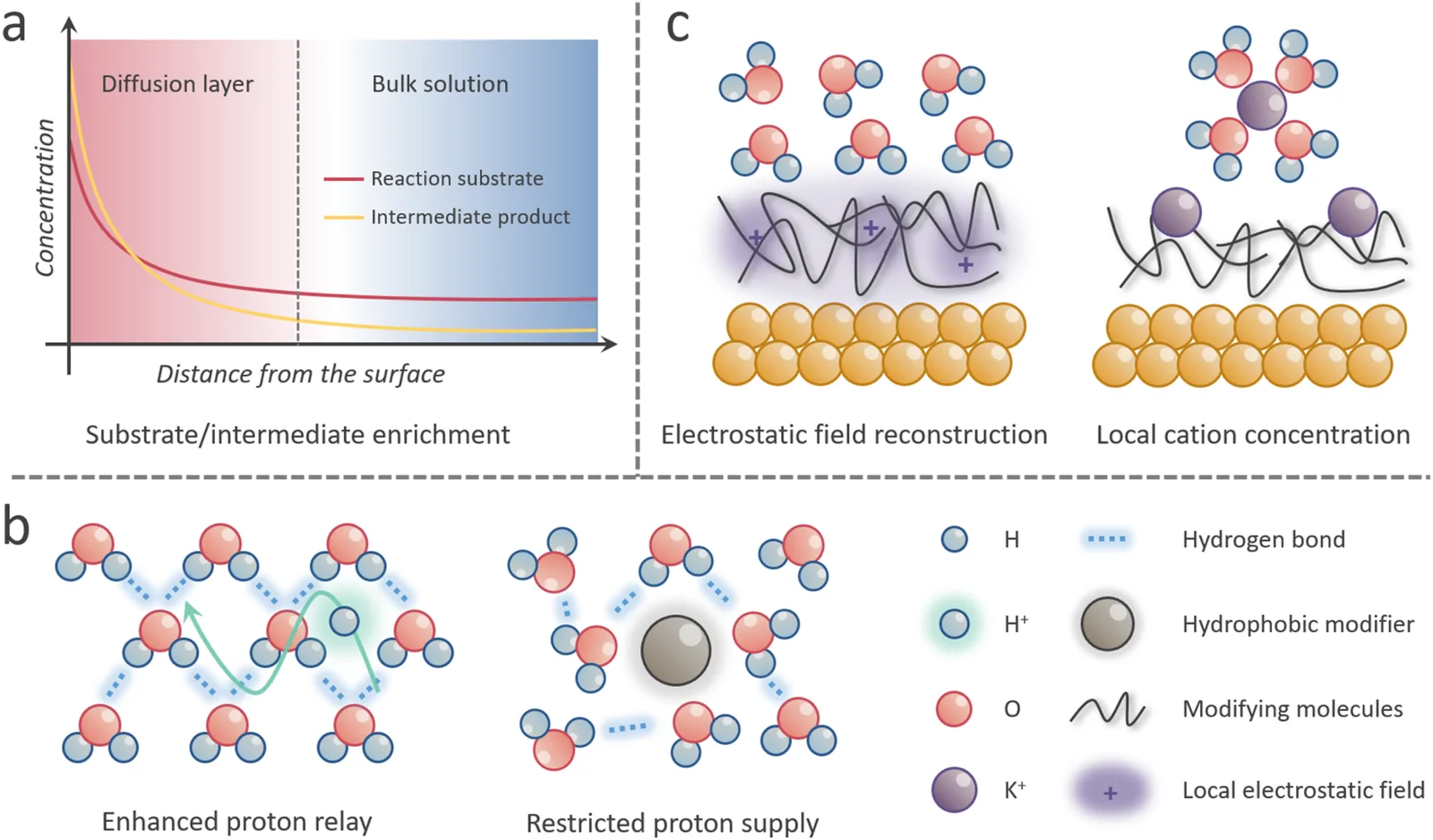
Intermediate-layer engineering for microenvironmental mediation. (a) Construction of local concentration fields to enrich specific substrates or intermediate products near the catalyst surface. (b) Reconfiguration of interfacial hydrogen-bond networks. Molecular modifiers can either construct ordered networks to facilitate rapid proton relay (left) or restrict interfacial water/proton supply to suppress competing side reactions (right). (c) Reshaping of the electrical double layer. The local interfacial potential and solvation structure can be optimized through direct electrostatic field reconstruction *via* charged groups (left) or indirect local cation enrichment *via* ion–dipole interactions (right).

The modifying molecules enrich reactants on the catalyst surface, generating a concentration higher than that in the bulk phase. Through weak interactions, modifying molecules can construct enrichment layers with specific affinity for target substrates, thereby mitigating limitations arising from low solubility or slow diffusion. As demonstrated by 4,4′-bipyridine modification on Ni(OH)_2_ enriches poorly soluble cyclohexanone near the surface and significantly increases adipic acid yield.^38^ Similarly, when 2,5-furandicarboxylic acid (FDCA) acts as a surface ligand, it can promote the near-surface accumulation of aromatic substrates through π–π stacking, thus enhancing both conversion efficiency.^39^ This enrichment effect also applies to ionic and gaseous substrates: polyaniline modification facilitates the co-enrichment of nitrate and reactive hydrogen species on CuO surfaces, whereas ethylene glycol modification improves CO_2_ accessibility near Pb interfaces.^40,41^ Collectively, concentration fields in the intermediate layer can be actively organized through molecular recognition and weak intermolecular interactions (Fig. 3a).

Beyond the primary reactants, tuning of local concentration fields is also critical for accumulating transient intermediates, particularly in multistep coupling reactions. In CO_2_ reduction systems involving C–C coupling, simply delivering reactants to the surface is insufficient; the key question is whether intermediates such as CO can be retained at the right location over an appropriate time scale. After Cu is modified with polyquinone layers, quasi-reversible chemical binding promotes CO_2_ activation and maintains high CO coverage, ultimately favoring C_2_H_4_ generation.^42^ Additionally, modifying a Cu surface with chlorin/hemin molecules containing Fe–N_4_ motifs mimics the natural CO-binding ability of enzymes, thereby enhancing CO adsorption and guiding subsequent multi-electron conversion.^43^ Concentration-field regulation changes the availability of species near the interface and indirectly affects subsequent electron transfer and intermediate coupling.

### 3.2 Hydrogen-bond networks

Whereas concentration fields mainly promote local substrate availability, hydrogen-bond networks affect proton transport more significantly. In electrochemical reactions, proton extraction is often associated with oxidation processes, whereas proton supply commonly accompanies reduction processes. The transport of protons across the electrode–electrolyte interface can therefore influence the overall reaction environment. Modulating the degree of ordering of the interfacial water network provides a means to regulate proton transport between vehicular diffusion and proton hopping (the Grotthuss mechanism).^44^ Hydrogen-bonding interactions and hydrophobic effects between interfacial H_2_O molecules and organic functional groups may affect the rate and directionality of local proton migration (Fig. 3b).^45^ Molecules bearing amino, carboxyl, or other hydrogen-bond donor/acceptor functionalities can help organize the interfacial water network, leading to more ordered proton-transfer pathways and improved proton transport. For example, amino-acid modification on CoP establishes a more concentrated proton reservoir near the surface, accelerates the PCET process, and improves HER performance.^46^ Consistently, terephthalate ligands on Co(OH)_2_ can rebuild hydrogen-bond networks in the double layer and function as proton relays, promoting lattice-hydroxyl activation and enabling ampere-level glycerol-oxidation currents.^47^

In some systems, however, the aim is to weaken proton migration. By disrupting the continuity of the water network, molecular modifiers can suppress proton-dependent competing reactions. After dimethyl sulfoxide (DMSO) adsorbs on Pt surfaces, interfacial water is confined into isolated nanoclusters, interrupting the long-range hydrogen-bond network. Once the local water cluster size falls below a threshold, interfacial proton transport becomes the rate-determining step, thereby suppressing the hydrogen evolution kinetics.^48^ Hydrophobic alkylthiols shield the cathodic field and drive interfacial water away from the easily dissociable H-down configuration, thereby regulating the proton supply to suppress hydrogen release during the carbon dioxide reduction process.^49^ Similarly, adsorption of butyltrimethylammonium bromide on Cu alters the double-layer structure, repels interfacial water, and weakens hydrogen bonding, thus suppressing hydrogen-atom-transfer pathways and shifting the product from furfuryl alcohol to the more valuable 2-methylfuran.^50^

The interplay between proton availability and HER suppression can exert a subtle influence on both reaction kinetics and product selectivity. In some cases, a moderated proton supply is often more advantageous than either unrestricted proton access or near-complete proton deprivation. For instance, in guanine-modified Cu, the modifier does not fully block proton transfer; instead, it maintains a relatively weakened but still operative proton-transport environment, making CO protonation and desorption to CH_4_ more favorable while suppressing the high CO coverage needed for C_2_ formation.^51^

Restricted proton transport can also prevent the rapid equilibration of proton consumption and generation at the interface, leading to local pH gradients. Such gradients enable distinct reaction steps to proceed under different local environments. For instance, in the electrochemical conversion of levulinic acid (LA) to γ-valerolactone (GVL), LA hydrogenation is favored under neutral or alkaline conditions, whereas the cyclization of the intermediate 4-hydroxypentanoic acid (HVA) requires strong acidity. By disrupting the interfacial hydrogen-bond network, cetyltrimethylammonium bromide (CTAB)-modified Pb electrodes limit proton transport and establish a local pH gradient, with a relatively alkaline electrode surface and an acidic bulk electrolyte. This spatially separates the tandem reaction sequence and enhances GVL production.^52^

These examples demonstrate that hydrogen-bond network mediated proton transport governs reaction pathways and catalytic selectivity by regulating proton availability and flux. Through such modulation, molecular modifiers can reshape local reaction environments and steer catalytic outcomes.

### 3.3 Interfacial electrostatic fields

Regulation within the intermediate layer can also be achieved through modulation of the interfacial electric field, thereby altering the local reaction environment (Fig. 3c). On the one hand, charged groups, molecular dipoles, or the modifiers themselves can directly redistribute the interfacial potential. On the other hand, the selective accumulation of electrolyte ions through ion-dipole or ion-binding interactions can indirectly alter the local potential distribution. Both effects contribute to the establishment of an electrostatic environment near the surface that differs from that of the bulk electrolyte.

Charged groups, dipole arrangements, or local cation layers introduced by molecular adlayers often establish electrostatic environments near the surface that differ from those in the bulk phase. As an example, polyelectrolytes bearing anchored piperidinium cations (Pip^+^) form stable, high-density local cation layers on Cu surfaces, modify the potential distribution, and simultaneously promote CO_2_ reduction while suppressing hydrogen evolution.^53^ Likewise, thiol modification at Cu interfaces can generate a spatial potential field that preferentially drives asymmetric coupling between adsorbed *COH and *CO, thus bypassing the high barrier associated with the conventional *CO dimerization pathway.^54^

Electrolyte cations can also be selectively enriched through molecular functional groups, consequently indirectly reconstructing the local solvation structure and ion transport. For instance, ethylene-glycol-functionalized poly(3,4-ethylenedioxythiophene) (PEDOT) coatings exploit strong interactions between EG side chains and alkali-metal cations to induce local ion enrichment, lowering the water-dissociation barrier and enhancing the release of reactive hydrogen.^55^ Cucurbit[6]uril can selectively bind K^+^ through ion-dipole interactions at its carbonyl portals and alter both the water structure in the double layer and the migration mode of OH^−^.^56^ Here, the role of the adlayers is not primarily to directly provide charge, but rather to indirectly tune the interfacial field and solvation network by capturing specific cations. Such cation-enrichment effects can be especially important under extreme conditions. Hydroxyethanediphosphonic acid (HEDP)-modified Cu_2_O can generate a K^+^-rich layer near the surface, partly shielding proton attack under acidic or low-K^+^ conditions while maintaining a high local pH environment more favorable for C–C coupling.^57^

In the intermediate layer, the core function of molecular engineering is thus modulating the microenvironment surrounding active sites. Concentration fields, hydrogen-bond networks, and electrostatic effects together constitute the main organizational principles of this layer. Acting collectively, they form an intermediate regulatory zone that bridges intrinsic catalytic activity and the macroscopic environment, making the intermediate layer a critical stage at which molecular modification advances from isolated activity intensification toward system-level performance optimization.

## 4. Outer-layer engineering: mass gating and interfacial protection

The outer layer molecular generally faces the bulk electrolyte directly and achieves long-term operational stability mainly by governing gas/liquid transport and enhancing interfacial robustness. Although this layer does not directly contact active sites, it more strongly affects device performance. When the outer-layer mass transfer, solvent accessibility, and structural stability are well matched, the advantages gained through inner-layer regulation are expected to be fully realized under high current density and long-term operation.

### 4.1 Wettability control

**Fig. 4:**
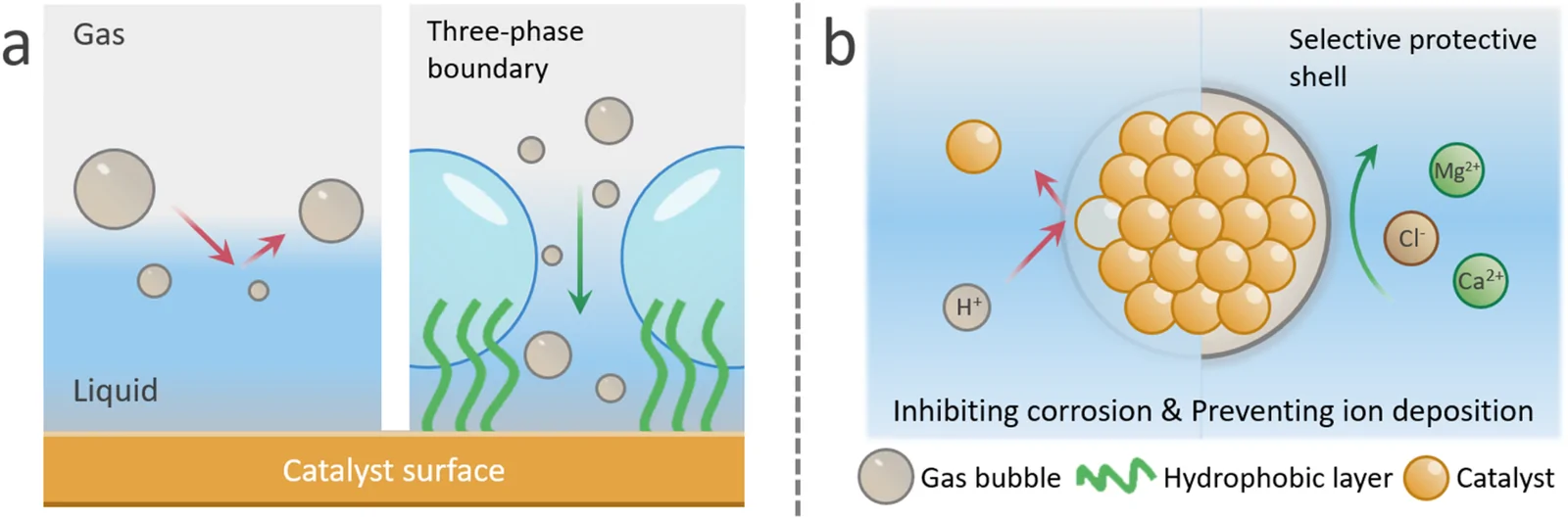
Outer-layer engineering for mass gating and interfacial protection. (a) Wettability control and mass transfer. Hydrophobic molecular layers prevent severe interfacial flooding and construct stable gas–liquid–solid three-phase boundaries, thereby significantly enhancing gas reactant accessibility. (b) Interfacial shielding and structural durability. Robust outer molecular shells act as selective physical/electrostatic barriers that repel corrosive anions and scaling-prone cations, preventing metal dissolution and ensuring long-term structural stability preservation under harsh conditions.

Tuning of interfacial wettability first affects the rate of species diffusion in multiphase reactions. In gas-involving processes such as CO_2_ reduction or N_2_ reduction, hydrophobic modification often reduces the excessive approach of H_2_O and protons to the surface while improving the accessibility of gas molecules at the macroscopic interface (Fig. 4a). For example, polytetrafluoroethylene (PTFE)-modified Cu surfaces construct a more hydrophobic three-phase boundary, suppress the competing hydrogen-evolution reaction, and elevate C_2_H_4_ selectivity.^58^ Following the mechanism, in N_2_ reduction, where reactant solubility is extremely low, the introduction of hydrophobic molecules such as organosilicon layers, CTAB, or fluorinated silanes facilitates water repellence at the interface and is accompanied by increased faradaic efficiency (FE) and NH_3_ production rate.^59,60^ However, overly strong solvent exclusion may cause local proton deficiency and increase charge-transfer resistance, thereby slowing certain subsequent hydrogenation steps. Designs of this type therefore require a balance between substrate enrichment and the proton supply needed for the desired pathway.

In liquid-phase organic electrocatalysis, wettability control acquires a somewhat different meaning. Here, the key issue is no longer the construction of a typical gas–liquid–solid three-phase boundary, but rather adjustment of surface hydrophilic/hydrophobic characteristics to facilitate transfer of hydrophobic organic substrates from the aqueous phase to the catalyst surface. For instance, sodium dodecyl sulfate (SDS) modification on Ni(OH)_2_ changes the surface wettability and markedly enhances interactions with hydrophobic cyclooctene, thus facilitating the diffusion and adsorption of organic reactants toward the anode and improving epoxidation efficiency and selectivity.^61^ During electrocatalysis, the molecular layer can undergo reconstruction due to applied voltage, local pH, and solvent conditions, leading to variations in interfacial wettability. On octadecanethiol (ODT)-modified Cu surfaces, the initially hydrophobic layer undergoes Cu-thiolate formation and alkylsulfonate dissolution during reaction, eventually evolving into a more stable three-phase interface accompanied by lower charge-transfer resistance and higher current density.^62^

### 4.2 Interfacial shielding

A second major role of the outer layer is to preserve catalyst structure under complex operating environments. Here, the molecular layer acts as a selective barrier (Fig. 4b): it is used to suppress deactivation pathways such as dissolution, aggregation, and corrosion without excessively sacrificing the transport of reactants or electrons. For instance, introducing organic ligand shells onto CoO_*x*_ surfaces can effectively mitigate the loss of catalyst dissolution during neutral water oxidation, thereby suppressing performance degradation.^63^ Similarly, polyaniline (PANI) can form a stable core–shell structure on Pt/C, where the shell physically isolates Pt nanoparticles from the corrosive electrolyte, suppresses Pt dissolution and migration, and mitigates oxidation of the carbon support. As a result, the catalyst exhibits improved stability as a cathode for proton-exchange membrane fuel cells under high-potential conditions.^64^

In highly corrosive electrolytic environments, such as natural seawater, molecular functionalization shifts from a structural stabilizer to a defensive barrier that resists corrosive anions and salt precipitation, thereby enabling long-term operation.^65^ As a case in point, 2-bromothiophene modification on Ni_3_N can simultaneously suppress halide corrosion and alkaline-earth-metal deposition while maintaining high H_2_O_2_ selectivity and long-term operational stability.^66^ Further, phytic-acid modification on Ni-based catalysts can form a PO_4_^3−^-containing layer that electrostatically repels chloride and facilitates surface reconstruction into the active NiOOH phase, supporting seawater oxidation at industrial current densities.^67,68^ These results suggest that in high-salinity and highly corrosive systems, molecular modifiers do not necessarily enhance activity directly, but they often determine whether the material can remain operational under complex conditions.

Overall, outer-layer engineering represents the most distal manifestation of molecular regulation at the catalyst–electrolyte interface. Rather than directly modulating intrinsic energetics, it governs mass transport, interfacial wettability, and structural robustness. This role becomes increasingly critical under high current densities, where diffusion limitations and degradation pathways dominate system performance. Consequently, the outer layer serves as a decisive factor that determines whether molecular engineering strategies can be effectively implemented and sustained under realistic operating conditions.

## 5. Mechanistic attribution and molecular design

The spatial classification of modifier effects established in the preceding sections provides a conceptual foundation for understanding how molecular modifiers regulate electrocatalytic interfaces. Building upon this framework, the following discussion addresses two complementary aspects: mechanistically attributing modifier effects to distinct interaction regions and translating these mechanistic insights into function-oriented molecular design principles.

### 5.1 Multi-layer attribution of modifier effects

A central challenge in molecularly modified electrocatalytic systems is that similar macroscopic catalytic responses often emerge from fundamentally different mechanisms operating across distinct spatial regions. Improvements in activity, selectivity, or stability may originate from direct electronic interactions at catalytic sites, reconstruction of the interfacial microenvironment, or modulation of mass transport. Reliable mechanistic understanding requires linking experimental observables and theoretical descriptors to the spatial region in which the underlying interactions predominantly operate (Table 2).

**Table 2:** Mapping of characterization and computational tools onto distance-aware interfacial regions

Category	Technique	Inner-layer region	Intermediate-layer region	Outer-layer region
Electronic and structural characterization	XPS/UPS	Core-level shifts, work-function variation	—	—
	*In situ* XAS/EXAFS	Coordination environment, oxidation state	—	—
*In situ* vibrational spectroscopy	*In situ* ATR-SEIRAS	Adsorbate structure, intermediate evolution	O–H stretching features, interfacial water structure	—
	*In situ* Raman	Surface species and structural evolution	Solvation-related spectral features	—
Electrochemical characterization and imaging	EIS	Charge-transfer kinetics	Relaxation processes and transport response	Mass-transport impedance
	SECM	—	Local concentration and pH distribution	Diffusion-layer evolution and reactant transport
Macroscopic interfacial properties	Contact angle/wettability	—	—	Wettability and three-phase interface behaviour
	Long-term electrolysis + XPS/ICP-MS	—	—	Surface composition and elemental stability
Multiscale computation	DFT	Adsorption energetics, electronic descriptors	—	—
	AIMD	Dynamic adsorbate evolution	Hydrogen-bond dynamics, solvent fluctuations	—
	MD	—	Interfacial density profiles, *g*(*r*)	MSD, diffusion behaviour
	Continuum modeling	—	Potential distribution, ion concentration profiles	Species flux, multiphase transport

Within the inner layer, modifier effects originate primarily from direct interactions with catalytic sites, including charge redistribution and orbital coupling. Accordingly, characterization focuses on electronic structure, local coordination, and adsorbate behaviour. X-ray photoelectron spectroscopy (XPS), ultraviolet photoelectron spectroscopy (UPS), *in situ* X-ray absorption spectroscopy (XAS), and extended X-ray absorption fine structure (EXAFS) provide information on electronic and structural modifications at the catalyst surface.^69^ Density functional theory (DFT) calculations quantify adsorption energetics, charge transfer, and electronic-state evolution. In addition, *in situ* vibrational spectroscopies, such as ATR-SEIRAS and Raman spectroscopy, monitor the evolution of adsorbed intermediates and surface-species coverage.

Within the intermediate layer, dominant effects arise from regulation of the electrochemical microenvironment, including solvent organization, hydrogen-bond networks, and local electric fields. Characterization therefore shifts toward probing interfacial water structures and local reaction environments. *In situ* vibrational spectroscopies reveal solvation structures and hydrogen-bond dynamics, while electrochemical impedance spectroscopy (EIS) and scanning electrochemical microscopy (SECM) provide insights into interfacial relaxation processes, concentration gradients, and local pH variations. In terms of theoretical simulation, *ab initio* molecular dynamics (AIMD) captures solvent fluctuations and interfacial dynamics at the atomic scale, whereas continuum modelling, often solved using finite element methods (FEM), describes electrostatic potentials and ion-distribution characteristics by treating the electrolyte as a continuous medium.^70^

Within the outer layer, modifier effects become increasingly associated with reactant transport, product removal, wettability, and interfacial stability. Relevant observables therefore include diffusion behaviour, mass-transport characteristics, and structural durability. EIS and SECM evaluate transport limitations and diffusion-layer evolution, while dynamic contact-angle measurements characterize wettability at the three-phase interface.^71^ Long-term electrolysis combined with post-reaction XPS and Inductively coupled plasma mass spectrometry (ICP-MS) analysis assesses modifier retention and catalyst degradation. Molecular dynamics (MD) and continuum models further provide quantitative descriptors of diffusion and multiphase transport processes.

By assigning experimental observables and theoretical descriptors to specific interaction regions, multi-layer attribution helps distinguish the dominant physicochemical origins of modifier effects and provides a conceptual basis for understanding how molecular engineering regulate catalytic performance.

### 5.2 Function-to-region design principles

**Fig. 5:**
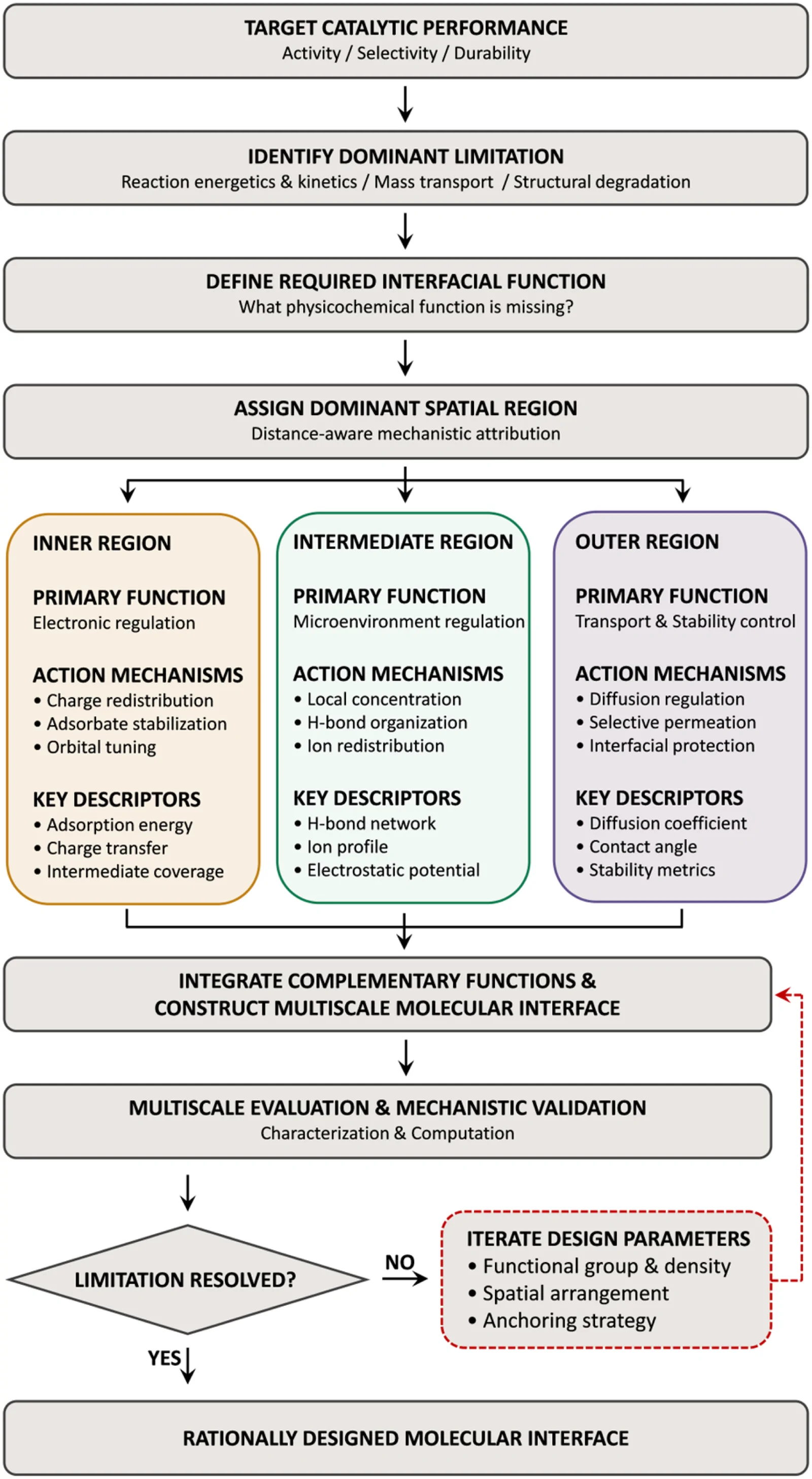
Workflow for the rational design of multi-layer molecular interfaces. This problem-driven framework integrates spatial functional assignment with an iterative evaluation loop to systematically optimize molecular parameters and achieve targeted catalytic performance.

Translating spatial attribution into design guidance involves correlating the required interfacial function with different regions of the molecular architecture. Within the distance-aware framework, distinct structural components of a modifier, such as the anchoring group, molecular backbone, and terminal functional moieties, may contribute preferentially to different spatial regions and therefore represent useful design variables for addressing specific performance limitations (Fig. 5).

Inner-region optimization (electronic and energetic limitations). In principle, when performance limitations are associated with intermediate stabilization or intrinsic reaction kinetics, molecular screening could focus on anchoring chemistry and local electronic effects. Relevant design variables include the nature of the anchoring atoms (*e.g.*, thiols, amines, or phosphonates), which can influence orbital interactions with the catalyst surface, as well as electron-donating or electron-withdrawing substituents positioned near the anchoring site to modulate work functions and adsorption energetics.

Intermediate-region optimization (microenvironment limitations). When catalytic behaviour is governed by proton availability, local pH, or solvent reorganization, greater attention may be placed on functional groups extending into the electrochemical double layer. Design considerations in this region include the density of hydrogen-bond donors and acceptors (*e.g.*, hydroxyl groups or pyridinic nitrogen species), localized charge states capable of influencing ion distributions, and steric configurations that shape local electrostatic potentials and concentration fields.

Outer-region optimization (transport and stability limitations). For systems limited by reactant diffusion, gas-bubble accumulation, or catalyst degradation, molecular design may focus on the molecular backbone and higher-order interfacial organization. Potential design variables include alkyl or fluorinated chain lengths that influence hydrophobicity and aerophilicity, steric environments that affect free volume for diffusion, and cross-linking characteristics that contribute to interfacial robustness and long-term stability.

By connecting dominant spatial limitations with relevant molecular design variables, the framework helps reduce the complexity of the molecular design space and provides a more structured basis for molecular screening. For example, acidic electrolytes can effectively suppress salt precipitation but simultaneously increase proton availability, which may accelerate catalyst degradation and intensify competition from the hydrogen evolution reaction. In such cases, the framework suggests complementary regulation across different spatial regions, Inner-layer engineering may enhance intrinsic catalytic activity or tune hydrogen adsorption energetics to improve proton utilization and corrosion resistance, whereas intermediate-layer engineering may regulate local proton concentration and transport through hydrophobic molecular moieties or electrolyte-affinitive functional groups that disrupt continuous hydrogen-bond networks. Extended molecular architectures may further contribute to stabilizing the outer three-phase interface and facilitating mass transport. Such examples illustrate that molecular engineering is inherently flexible and multifaceted; rather than prescribing universal design rules, the framework helps prioritize promising molecular engineering strategies and narrow the experimental search space according to the dominant catalytic limitations.

## 6. Cross-layer synergy and application boundaries

Dividing the electrocatalytic interface according to distance helps distinguish different origins of tuning, including electronic tuning, microenvironment shaping, and macroscopic mass transfer. In real systems, however, these processes are rarely independent. The most significant performance improvements associated with molecular engineering generally arise from coordinated orchestration across different spatial layers. After decoupling the effects through a distance-aware perspective, a cross-layer view enables a more unified understanding of molecular engineering.

### 6.1 Cross-layer synergistic systems

Although modifier-induced effects can be conceptually decoupled into distinct spatial regions, practical molecular interfaces often operate through cascading or coupled interactions across multiple spatial layers. Such multidimensional regulatory modes may range from dual-layer coupling to fully integrated three-layer synergy, collectively enhancing catalytic activity, selectivity, and stability.

Synergy between the inner and intermediate layers typically combines direct electronic activation with reinforcement of the local concentration field. For example, in Cu-based catalytic systems, a dipole-rich camphor-derived polymer simultaneously regulates both regions. At the inner layer, its sp^3^-hybridized nitrogen atoms act as Lewis bases, inducing surface charge redistribution that alters CO adsorption configurations and lowers the barrier for C–C coupling. At the intermediate layer, its strong dipole moment polarizes and restructures the inner Helmholtz plane, leading to a fourfold increase in the local CO_2_ concentration.^72^ Similarly, self-assembled thiol monolayers can generate localized electrostatic fields that lower the surface work function in the inner layer, thereby facilitating spontaneous CO dimerization, while simultaneously displacing interfacial water molecules in the intermediate layer and substantially expanding the CO_2_-enriched boundary region.^54^

Conversely, coupling intermediate-layer microenvironment regulation with outer-layer macroscopic protection is particularly important for maintaining catalyst stability under harsh operating conditions, such as strongly acidic electrolytes or low-CO_2_ feed concentrations. Coating Cu surfaces with imidazolium-based ionomers can establish a proton-blocking layer within the intermediate region, suppressing the hydrogen evolution reaction while enriching CO_2_ through weak molecular interactions. At the outer layer, the hydrophobic nature of the coating stabilizes the gas–liquid–solid three-phase interface and mitigates electrode flooding, enabling sustained high C_2+_ selectivity even under strongly acidic conditions.^73^ A similar strategy has been demonstrated in Ni single-atom catalysts modified with covalently grafted hydrophobic alkyl chains. In this case, the alkyl chains disrupt the interfacial hydrogen-bond network in the intermediate layer, suppressing water dissociation, while simultaneously imparting super aerophilic properties at the outer layer, greatly enhancing underwater CO_2_ bubble adhesion and macroscopic interfacial stability.^74^

A more ideal interfacial engineering design aims to achieve synergistic cooperation across all three spatial layers. Notably, natural biopolymers such as chitosan and cellulose can function as versatile modifiers spanning the entire interface. At the inner layer, they interact strongly with Cu surfaces to stabilize favorable *CO adsorption configurations and promote C–C coupling. At the intermediate layer, they reorganize the interfacial water network, increasing local pH and ionic conductivity. At the outer layer, they act as environmentally benign binders free of polyfluoroalkyl substances (PFAS), maintaining structural integrity while preserving interfacial hydrophilicity. This integrated regulation enables exceptional C_2+_ production rates at industrially relevant current densities, demonstrating that hydrophilic materials can simultaneously achieve efficient mass transport and long-term operational stability.^75^

Collectively, these progressively integrated coupling modes illustrate that high-performance catalytic interfaces do not arise from the simple superposition of isolated mechanisms but rather from continuous, multidimensional regulatory networks operating across spatial boundaries.

### 6.2 Boundaries and trade-offs

Although molecular engineering provides a highly flexible toolset for interfacial modulation, attribution of performance enhancement requires caution. One phenomenon that deserves particular attention is that, in some molecularly functionalized Cu catalytic systems, the intrinsic activity normalized by electrochemically active surface area does not increase and may even fall below that of the unmodified reference. This suggests that observed changes in FE and product selectivity do not necessarily originate from enhanced intrinsic activity at reaction sites, but may instead be associated with changes in gas–liquid diffusion rates or local concentration fields.^76^ Consistently, on-chip microdevice platform used to decouple the effect of methylene blue (MB) on MoS_2_, it is found that although MB incorporation increases the charge carrier density, this is not the dominant factor for the enhanced HER activity. On-chip electrochemical impedance spectroscopy confirms that the true driving force for this performance enhancement is the local proton enrichment induced by the nucleophilic groups of MB.^77^

The coverage of the molecular layer also defines the operative window of its effect. Taking the octylamine shell on Cu_2_O as an example, a moderate shell thickness helps delay desorption of key intermediates and improves C_2+_ selectivity. However, when the shell becomes too thick, molecular packing becomes dense and both mass transfer and charge transfer are significantly hindered; when it is too thin, the layer is insufficient to provide effective stabilization.^78^ Introducing molecular modifiers maybe imposes additional diffusion or transport costs, meaning that catalyst design proceed through careful trade-offs.

A key limitation arises from the chemical stability of molecular layers themselves. Some modifiers are anchored to catalyst surfaces through relatively weak interactions, and ligand detachment may accompany operation. Under strongly reducing potentials, thiol ligands on Au nanoparticles detach almost completely, whereas N-heterocyclic carbenes can remain at relatively high coverage and continue to exert regulatory effects.^79^ Many common organic ligands, including amines, amides, phosphines, polycyclic/heteroaromatic compounds, and isocyanides, can remain relatively stable under strongly reducing environments, but are more susceptible to bond cleavage, oxidation, or hydrolysis under strongly oxidative conditions, particularly within the oxygen evolution reaction (OER)-relevant potential window.^80^ This limitation on the transferability of molecular engineering implies that molecules exhibiting good performance in cathodic systems should be transferred to anodic systems with caution.

## 7. Summary and outlook

This perspective provides a systematic distance-aware framework to decouple and integrate the multifaceted modulatory effects of molecular engineering at electrocatalytic interfaces. This framework helps to organize understanding of the systematic variations and intrinsic links in molecular functionalization effects with respect to spatial distance from the active site. Inner-layer regulation operates in the immediate vicinity of the active site and therefore directly modulates the intrinsic energetics of the catalyst. It typically exerts the most pronounced impact on catalytic activity, albeit with the highest degree of structural sensitivity. Intermediate-layer regulation mediates the competition among distinct reaction pathways by structuring the local microenvironment, including concentration gradients, hydrogen-bonding networks, and the electric double layer, thereby representing a versatile and broadly transferable strategy. In contrast, outer-layer regulation, while spatially distant from the active centre, plays a decisive role in preserving and enabling the effective manifestation of the advantages imparted by the inner and intermediate layers during catalysis. Importantly, these roles are not mutually isolated in practical systems. From a distance-aware perspective, molecular engineering can be understood as a spatially continuous and hierarchically coupled mode of interfacial modulation. This physically grounded framework facilitates the distillation of common principles from fragmented experimental observations. Building on this framework, several research directions warrant further exploration and attention.

### 7.1 From characterization to mechanistic discrimination

Although extensive mechanistic studies have substantially advanced the understanding of molecular engineering, the contributions of coupled regulatory effects remain difficult to disentangle, leaving multiple mechanistic interpretations plausible. A critical future direction is therefore to resolve these open questions through *operando* identification and dynamic tracking of interfacial states under realistic reaction conditions. Advances in *operando* characterization and multiscale theoretical modelling will be essential for distinguishing competing mechanisms and deepening our understanding of molecular engineering.

### 7.2 Developing adaptive molecular interfaces

Traditional modifying molecules often suffer from instability, functional degradation, and mass transport limitations under long-term operation or harsh conditions, and their transferability between different systems is also poor. To address these issues, developing dynamic interfaces that can respond to changes in electrical potential, pH, local concentration, or temperature offers a promising strategy to transform the interface from a passive barrier into an active functional unit. Such dynamic interfaces enable reversible regulation, self-repair, and interfacial renewal during operation, thereby overcoming the inherent limitations of static molecular coatings.

### 7.3 Leveraging machine learning and data-driven design

As the molecular design space and interfacial complexity continue to expand, machine learning and data-driven approaches are poised to play an increasingly pivotal role in rational molecular engineering. Future efforts could focus on integrating molecular descriptors, interfacial interaction features, and electrocatalytic performance into unified datasets, thereby, which could ultimately support the development of multiscale predictive models for efficient screening of modification strategies across vast candidate spaces.

Overall, the distance-aware framework not only provides a perspective for interpreting the relationships among diverse molecular regulation mechanisms, but also offers a structured framework for thinking about the rational design of future electrocatalytic interfaces. With continued progress in cross-layers coupling, dynamic interfaces, and data-driven methods, molecular engineering may gradually evolve from an empirical optimization strategy into an approach with stronger predictive and designable aspects.

## Author contributions

Z. Wang: writing – original draft, conceptualization, investigation, formal analysis, methodology. X. Zhang: investigation, formal analysis. L. He: formal analysis, validation. Z. Chen: writing – review and editing, conceptualization, supervision, funding acquisition, resources. Z. Ren: writing – review and editing, conceptualization, resources, project administration, funding acquisition.

## Conflicts of interest

There are no conflicts to declare.

## Data Availability

No primary research results, software or code has been included and no new data were generated or analyzed as part of this perspective.
